# Spontaneously Hypertensive Rats Present Exacerbated Focal Stroke Behavioral Outcomes

**DOI:** 10.3390/brainsci14080838

**Published:** 2024-08-21

**Authors:** João Victor Matos e Moreira, Luis Pedro Bernardi, Fernanda Cardoso Teixeira, Jerônimo Paniago, Luciele Varaschini Teixeira, Felippo Bifi, Diogo Onofre Souza, Francieli Rohden

**Affiliations:** 1 Graduate Program in Biological Sciences: Biochemistry, Universidade Federal do Rio Grande do Sul, Annex Building, Ramiro Barcelos Street 2600, Porto Alegre 90035-003, Rio Grande do Sul, Brazil; victormatosmoreira@gmail.com (J.V.M.e.M.); luispedrobernardi2@gmail.com (L.P.B.); paniagojeronimo@gmail.com (J.P.); luciele.sm@gmail.com (L.V.T.); felippo.96@gmail.com (F.B.); diogo@ufrgs.br (D.O.S.); 2 Graduate Program in Biosciences, Federal University of Health Sciences of Porto Alegre—UFCSPA, Porto Alegre 90050-170, Rio Grande do Sul, Brazil; fe.t@hotmail.com

**Keywords:** ischemic stroke, memory, rats, sensorimotor, systemic arterial hypertension

## Abstract

This study aimed to analyze the effects of systemic arterial hypertension (SAH) in a model of permanent ischemic stroke (focal ischemia due to thermocoagulation of pial vessels) on sensorimotor function (cylinder test and patch removal test), behavioral tasks (novelty habituation memory open field task) and cerebral infarct size in adult male spontaneously hypertensive rats (SHR) and normotensive Wistar Kyoto rats (WKY) for 42 days after the occurrence of a stroke. We observed that the stroke caused asymmetry in the front paws and delayed adhesive removal. These effects were spontaneously reduced in WKY rats, but not in SHR. Short- and long-term novelty habituation memories were abolished by stroke in WYK and SHR. On the 3rd day after stroke, the size of the focal cerebral infarct was the same in WKY and SHR. However, on the 7th day, the infarct size decreased in WKY rats, but not SHR. These results suggested that SAH impairment of sensorimotor recovery in rats subjected to cerebral ischemia could be related to augmented focal cerebral infarct size. Moreover, the behavioral tasks used in this study were unaffected by Systemic Arterial Hypertension. Our results highlight the need for animal models of comorbidities in stroke research.

## 1. Introduction

### Stroke

Approximately 17 million individuals worldwide experience stroke each year, which is a leading cause of adverse outcomes and mortality [1,2,3,4]. Stroke is categorized into two main types: hemorrhagic and ischemic strokes [5,6]. Ischemic stroke is the most prevalent type, accounting for approximately 80 percent of all stroke cases [7,8].

In the brain affected by ischemic stroke, the central area of damage, known as nuclear infarction, causes cell death and irreversible tissue lesions [9,10]. The region surrounding the nucleus infarction, referred to as the penumbra in humans or the peri-infarct zone in animals, is affected, but can potentially recover. Recovery from this region is the primary goal of ischemic stroke treatment strategies for ischemic stroke [11]. Delays in intervention can lead to poor patient outcomes [12,13,14].

Systemic arterial hypertension (SAH) is a multifactorial medical condition characterized by a persistent increase in blood pressure, affecting several individuals worldwide [14,15,16]. SAH causes mechanical stress on the arterial walls, resulting in remodeling of the neurovascular structure and cerebral blood flow [17]. Thus, SAH is the main risk factor for stroke [14,18] and contributes to worse outcomes [19,20,21,22]. As most preclinical studies on stroke have been conducted on healthy young animals, it is essential to develop studies that consider age and relevant comorbidities, such as SAH [13,23]. 

Spontaneously Hypertensive Rat (SHR) [23,24,25,26], in an animal model, is well-established for studying systemic arterial hypertension This model, developed through selective breeding of Wistar Kyoto (WKY) rats, is a robust genetic model for hypertension [24,25] that serves as the homologous control group for SHR [26,27].

This study evaluated the behavioral outcomes produced by an experimental model of ischemic stroke caused by thermocoagulation of pial vessels in hypertensive (SHR) and normotensive (WKY) rats by examining sensorimotor function, short- and long-term memory, and brain lesion size. The results should contribute to the development of more effective clinical intervention strategies, considering aspects such as personalizing treatments to meet individual patient needs, improving diagnostic accuracy, and identifying new therapeutic approaches that could enhance treatment success rates and minimize side effects.

## 2. Materials and Methods

### 2.1. Animals

This study used male SHR and WKY rats aged—90–120 days obtained from the vivarium of the Experimental Cardiology Center of the Institute of Cardiology/University Foundation of Cardiology. The animals were kept at a temperature of 22 ± 2 °C in a controlled light ambiance (light/dark cycle of 12/12 h), with water and standard animal feed ad libitum. Animals were housed in polycarbonate boxes lined with wooden shavings.

The rats were divided into two main experimental groups: (1) hypertensive (SHR) and (2) normotensive (WKY). Each group was further subdivided into (1) animals subjected to ischemic stroke by thermocoagulation of the pial vessels (ISC), (2) animals subjected to craniectomy without ischemic stroke by thermocoagulation of the pial vessels (sham), and (3) controls without surgical intervention (naive).

### 2.2. Ethical Aspects

All procedures involving animals followed the guidelines recommended by the National Guidelines for Animal Experimentation, according to Brazilian law 11,794 of 10 August 2008 [28] and the policies of the National Council for the Control of Animal Experimentation (CONCEA) [29] and Guide for Care in the Use of Laboratory Animals from the National Institute of Health [30]. The animals were euthanized following Law 714, 20 June 2002, which provides procedures and methods for the euthanasia of animals and other measures of the Federal Council of Veterinary Medicine, Law 11,794 of 8 October 2008, and the Practice Guidelines of Euthanasia at CONCEA [31]. The authors declare that they followed the guidelines of the National Research Council’s Guide for the Care and Use of Laboratory Animals [32] and made every effort to minimize animal suffering and discomfort, using the fewest animals possible to obtain consistent results. If any discomfort or suffering was observed, we implemented a humane endpoint.

This study was approved by the Ethics Committee on Animal Use of the Institute of Cardiology/University Foundation of Cardiology and registered under protocols UP 5806/20 and UP 5850/20.

### 2.3. Focal Permanent Stroke by Thermocoagulation of Pial Blood Vessels

Stroke is induced by thermocoagulation of pial blood vessels [33,34,35,36,37]. The animals were anesthetized with ketamine hydrochloride (90 mg/kg, 450 µL/kg i.p.) and xylazine hydrochloride (10 mg/kg, 300 µL/kg i.p.) and then placed in stereotaxic apparatus. A craniectomy was performed by exposing the left parietal cortex [+2 to −6 mm AP (anteroposterior) and −2 to −4 mm ML (medium-lateral) from the bregma].

The pial vessels were thermocoagulated for 2 min using a warm probe. The skin was then sutured with a mono-nylon thread using simple isolated stitches and the animals were kept on a thermal mattress (37 °C) until recovery from anesthesia.

### 2.4. Sensorimotor Functions Evaluation

#### 2.4.1. Cylinder Test

Symmetry of the front paws was assessed using the cylinder test (CT) [36,37]. The animals were placed inside a glass cylinder with a diameter of 20 cm and a height of 30 cm. An observer recorded the first 20 touches made by the front paws of the animals on a cylindrical wall. These touches were categorized as ipsilateral, contralateral (relative to the side of the brain injury), or both. After each session, the apparatus was thoroughly cleaned with 70% alcohol.

The asymmetry for each animal was calculated using the formula: asymmetry = (% of ipsilateral paw touches = ipsilateral paw touches/total sum of touches) − (% of contralateral paw touches = % of contralateral paw touches/total sum of touches). Subsequently, asymmetry was converted to % symmetry (100% asymmetry) [36,38,39]. The animals were evaluated 1 day before surgery (day-1) and 3, 7, 14, 21, 28, 35, and 42 days after the stroke. The exclusion criterion for the animals was symmetry in the cylinder test. WKY animals with symmetry above 70% after 3 days of ischemia, and SHR after 14 days, were excluded from the study.

#### 2.4.2. Adhesive Removal Test

The adhesive removal test (ART) is one of the most efficient behavioral tests for identifying sensorimotor deficits [40,41]. To administer the test, a paper sticker with a diameter of 13 mm was attached to the underside of the front paws of each animal. The animals were then placed in an acrylic experimental box measuring 30 cm in length × 22 cm in width × 22 cm in height for 60 s. The time taken to remove the adhesive from each paw (referred to as removal latency) was recorded. The test was repeated five times with a five-minute interval between trials. To calculate the latency of adhesive removal for both the contralateral and ipsilateral paws, the average of the two shortest removal times was determined from five tests [41]. The animals were evaluated 1 day before surgery (day-1) and 3, 7, 14, 21, 28, 35, and 42 days after surgery.

### 2.5. Memory Evaluation—Open Field Task

This task was designed to evaluate short- and long-term novelty habituation memories. The animals were placed in a non-transparent black box measuring 50 cm length × 50 cm width × 50 cm height. The locomotor activity of the animals was recorded for 10 min using a camera installed above the box and the distance walked per minute was calculated using ANY-Maze software, Version 7.37 (Stoelting Co., Wood Dale, IL, USA). At the end of each session, the apparatus was cleaned using 70% alcohol. Evaluations were conducted 7 and 21 days after surgery.

Short-term memory is defined as a decrease in locomotion from the 1st to the 5th min of the first exposure, whereas long-term memory is defined as a decrease in locomotion in the 1st min of successive exposure [36,37].

### 2.6. Infarct Size Evaluation

The infarct size was calculated on the 3rd or 7th day post-surgery. Animals were anesthetized with ketamine hydrochloride (90 mg/kg, 450 µL/kg i.p.) and xylazine hydrochloride (10 mg/kg, 300 µL/kg i.p.). Subsequently, the brain was quickly removed from the skull, frozen, and sectioned in the coronal plane into slices of 2 mm thickness. The slices were immersed in a 2% solution of 2,3,5-triphenyl tetrazolium chloride (TTC), a red color dye, for 30 min at 37 °C, followed by fixation in 4% PFA solution for 24 h [35,36,37]. The slices were then placed in the dark, and images were captured. Areas without red coloration were considered necrotic (infarcted) and represented lesions. The total brain and lesion volumes were measured using ImageJ software 1.54j. The measurements were then used to calculate the percentage of lesion volume [36,37].

### 2.7. Statistical Analysis

We conducted a Shapiro–Wilk test as part of our statistical analysis to verify the normal distribution of the groups (Supplementary Materials). The effects of stroke on the behavioral parameters of the rats (TC, TRA, and OFT) were analyzed using a two-way ANOVA, followed by Sidak’s multiple comparisons. A two-way ANOVA followed by Sidak’s multiple comparison test was also used to compare brain lesion volumes between SHR and WKY rats over time. Data were expressed as mean ± SEM for behavioral assessments and mean ± SD for lesion volume. An alpha value (significance level) of 0.05 was used for all the tests. All analyses were performed using Graph Pad Prism 9.0 software. 104 animals were initially used in the study, but 102 remained at the end. Two animals were excluded: one WKY ISC 3 days and one WKY ISC 7 days, due to humane euthanasia. The animals in the ISC WKY and SHR groups (12 per group) were consistent across all three behavioral tests: CT, ART, and OFT. However, different animals were used for the Naive groups in the OFT compared to those used in the CT and ART tests for both WKY and SHR.

## 3. Results

### 3.1. Cylinder Test (CT)

**Figure 1:**
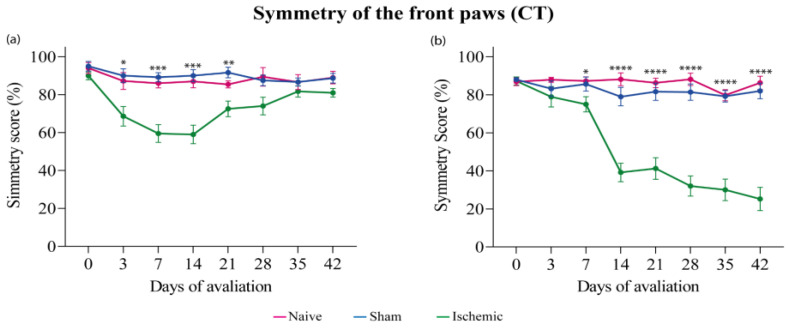
Symmetry of the front paws of WKY (**a**) and SHR (**b**) groups in the CT. Data expressed as mean ± SEM, analyzed by two-way ANOVA, followed by Sidak’s multiple comparisons test. The * *p* < 0.05, ** *p* < 0.01, *** *p* < 0.001, **** *p* < 0.0001, ISC groups compared to naive and sham groups. WKY groups: naive (n = 5), sham (n = 5), ISC (n = 12); SHR groups: naive (n = 5), sham (n = 5), ISC (n = 12).

A CT was performed one day before surgery (day-1) and once a week from the 3rd to the 42nd day (end of the experiment) post-surgery. The ISC WKY group exhibited a significant symmetry reduction in CT from the 3rd day that spontaneously recovered on the 21st day post-ischemia (Figure 1a). The ISC group exhibited a significant reduction only from the 7th day post-ischemia that was intensified until the end of the experiment (Figure 1b). No alterations in symmetry were observed in the naive or sham groups. Individual results are in the Supplementary Figure S1.

### 3.2. Adhesive Removal Test (ART)

**Figure 2:**
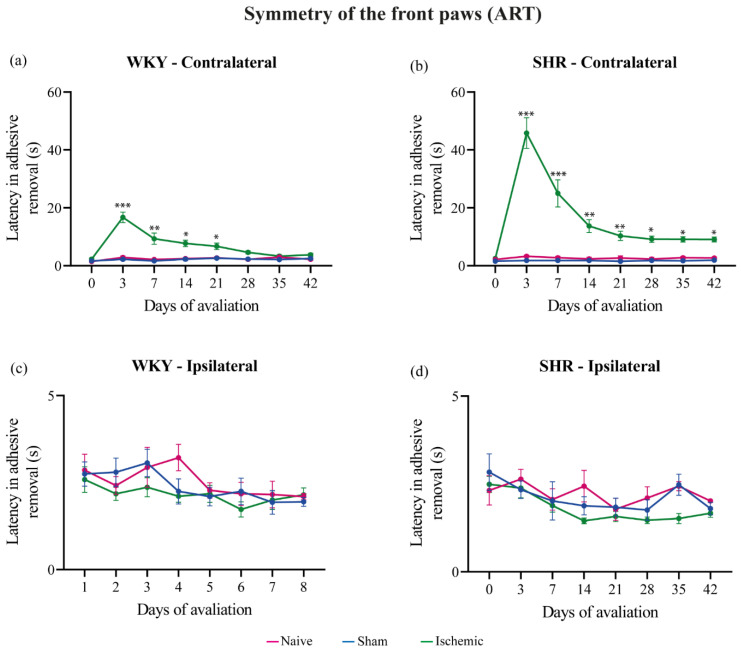
Latency of adhesive removal from front paws of WKY (**a**,**c**) and SHR (**b**,**d**) groups. Data expressed as mean ± SEM, analyzed by two-way ANOVA followed by Sidak’s multiple comparisons. The * *p* < 0.05, ** *p* < 0.01, *** *p* < 0.001, ISC groups compared to naive and sham groups. WKY: naive (n = 5), sham (n = 5), ISC (n = 12); SHR: naive (n = 5), sham (n = 5), and ISC (n = 12).

Adhesive removal of the ipsilateral and contralateral paws was evaluated on day-1 and once a week from the 3rd to the 42nd day post-surgery (end of the experiment). The ISC WKY and SHR groups exhibited a significantly prolonged adhesive removal latency from the contralateral paw on the 3rd day post-stroke. In the ISC WKY group, this effect spontaneously disappeared on the 28th day (Figure 2a), In contrast, in the ISC SHR group, the prolonged adhesive removal latency persisted until the end of the experiment (Figure 2b). No alterations in symmetry were observed in the naive or sham groups. Individual results are in the Supplementary Figure S2.

### 3.3. Open Field Task (OFT)

**Figure 3:**
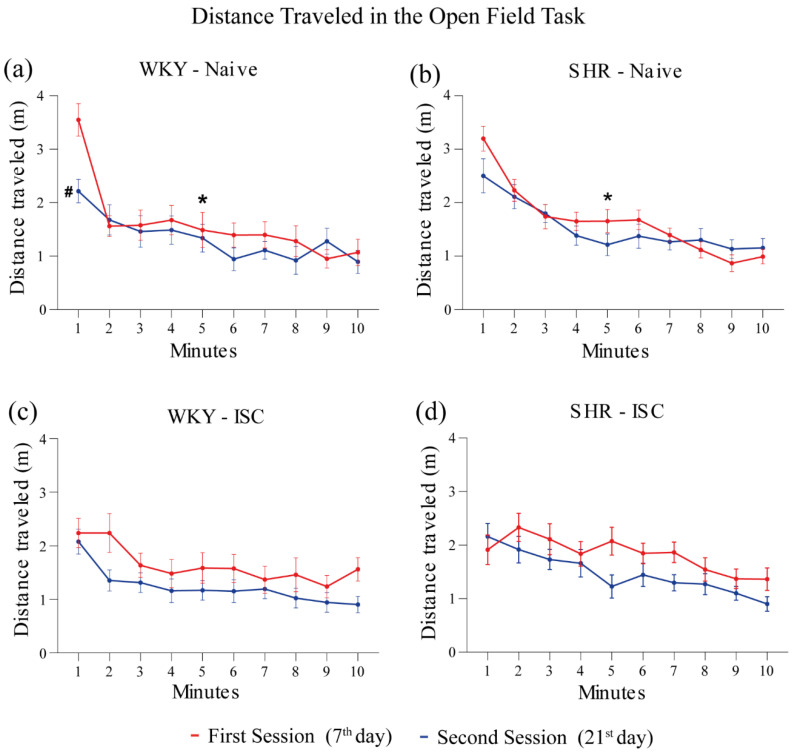
Distance traveled in the OFT on the 7th (first session) and 21st (second session) day after stroke. Naive WKY (**a**) and naive SHR (**b**) groups; ISC WKY (**c**) and ISC SHR (**d**) groups. Data expressed as mean ± SEM analyzed by two-way ANOVA followed by Sidak’s multiple comparisons. The * *p* < 0.05, comparing the 1st min with the 5th min in the first session (short-term memory); ^#^ *p* < 0.05, comparing the 1st min of the first session with the 1st min of the second session (long-term memory). Naive WKY (n = 12), Naive SHR (n = 12), ISC WKY (n = 12), and ISC SHR (n = 12).

The distance traveled in two successive sessions of the OFT is shown in Figure 3 for the WKY (Naive, ISC) groups and SHR (Naive, ISC) groups. The naive WKY (Figure 3a) and naïve SHR (Figure 3b) groups showed a significant decrease in the distance traveled in the first session from the 1st to the 5th minute, indicating short-term memory [42]. However, this difference was not observed in the ISC WKY (Figure 3c) and ISC SHR groups (Figure 3d), indicating that stroke affects the short-term memory of habituation to novelty [42]. Moreover, only naïve WKY rats (Figure 3a) presented long-term memory of habituation to novelty, as evidenced by a decrease in locomotion when comparing the 1st minute of the first session with the 1st minute of the second session, indicating that stroke and/or HAS affected the long-term memory of habituation to novelty.

### 3.4. Infarct Size

**Figure 4:**
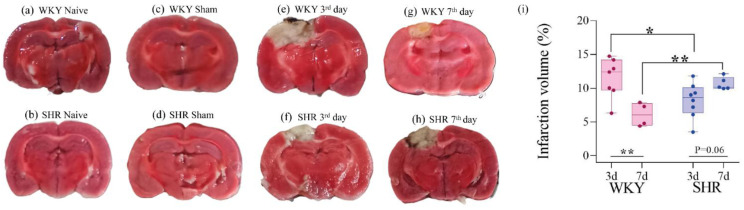
Infarct size in WKY and SHR. Representative images from brain slices stained with TTC (**a**–**h**). Comparison of infarct size on the 3rd day with the 7th day post-stroke in WKY and SHR (**i**). Data are expressed as mean ± SD, analyzed by two-way ANOVA, followed by Sidak’s multiple comparisons test. The * *p* < 0.05 and ** *p* < 0.01. Naive (n = 5), Sham (n = 5), WKY ISC 3 days (n = 7), WKY ISC 7 days (n = 4), SHR ISC 3 days (n = 8), and SHR ISC 7 days (n = 5).

On the 3rd day post-stroke, the infarct size in the ISC WKY group (Figure 4e) was significantly larger than that in the ISC SHR (Figure 4f). On the 7th day post-stroke, the infarct size in the ISC WKY (Figure 4g) group decreased compared to that on the 3rd day (Figure 4e), whereas in the ISC SHR group on the 7th day post-stroke, it remained the same as that on the 3rd day (Figure 4f,h). Statistical analysis of infarct size is shown in Figure 4i. No ischemic lesions were detected in the brains of WKY and SHR rats in either the naive or sham groups (Figure 4a–d). The smaller infarct size on the 3rd day in SHR compared to WKY rats could be correlated with the absence of asymmetry that was only observed in the SHR on the CT conducted on the 3rd day after stroke (Figure 1b).

## 4. Discussion

Animal models of cerebral ischemia are essential tools for understanding the pathophysiology of stroke and for developing new therapeutic and protective strategies. In the present study, we evaluated the effects of HAS on the outcome of a stroke model using rats’ spontaneous hypertension. In this study, we employed a permanent model of focal ischemic stroke through thermocoagulation of the pial vessels. This model, which is easily reproducible, is widely used in our research group [33,34,35,36,37] (REF). We assessed the following parameters in hypertensive rats (SHR) and normotensive rats (WYK): (i) loss in the recovery of post-ischemic sensorimotor function (CT and ART), (ii) impaired short- and long-term memory (OFT), and (iii) increased brain lesion size. 

The SHR group, subjected to a stroke, exhibited a decrease in the symmetry of their front paws, while the WKY group experienced spontaneous recovery. In both groups, these results persisted until the end of the experiment. These findings are consistent with those of previous studies using various cerebral ischemia models and/or different animal species, which consistently indicated greater neurological impairment in hypertensive rats than in normotensive rats [43,44]. Tchekalarova et al., 2023, demonstrated that WKY rats achieved full functional recovery much earlier than SHR [26]. Investigations by our group using the same stroke model in Wistar rats indicated the recovery of front paw symmetry up to the 42nd day post-stroke [36,37]. These findings highlight the divergent post-ischemic functional motor responses of Wistar rats compared to those of WKY and SHR rats, highlighting the importance of conducting studies with different strains and models of cerebral ischemic injury. 

A possible mechanistic explanation for this result in SHR rats involves several neurovascular changes, such as cerebral blood vessel remodeling, blood–brain barrier dysfunction, impaired regulation of cerebral blood flow, and functional hyperemia [17,45]. Furthermore, they often show increased neuroinflammation compared to normotensive rats. Chronic hypertension can exacerbate the activation of glial cells (microglia and astrocytes), leading to a more intense inflammatory response after ischemic events [46]. Hypertension can also increase the permeability of the blood–brain barrier and alter the function of neurotransmitters, resulting in increased release of excitotoxic neurotransmitters such as glutamate and increased activation of glutamatergic receptors. So, in SHR rats, excitotoxicity may be more pronounced due to changes in glutamate regulation and the central nervous system response to stress [47]. Furthermore, SHR rats may have compromised mitochondrial function and altered energy metabolism due to chronic hypertension. This may lead to a more severe response to ischemic stress, as their reduced ability to generate ATP and maintain cellular homeostasis increases neuronal vulnerability to damage [48]. Chronic hypertension may also predispose neurons to increased apoptosis. The combination of exacerbated neuroinflammation, increased excitotoxicity, and greater energy deprivation in SHR rats makes neurons more prone to programmed cell death. Following ischemia, the rate of apoptosis may be higher in SHR rats due to pre-existing adverse conditions that exacerbate the injury response [49]. Therefore, spontaneous hypertension in SHR rats may amplify the response to ischemia and other brain stresses through several mechanisms, including exacerbated neuroinflammation, increased excitotoxicity, increased energy deprivation, and increased apoptosis. These factors combined may result in more severe brain damage, hindering spontaneous recovery in SHR rats compared to normotensive models.

In this study, the assessment of short- and long-term memory revealed notable differences between WKY rats and SHR. The naive SHR group exhibited a deficiency in long-term memory in the OFT, which agreed with previous studies that have consistently suggested that hypertensive rats exhibit memory impairment compared to normotensive rats [50,51]. Even though the SHR animals did not present a long-term memory, we also observed that both groups of naive animals covered the same distance in the first minute of the first exposure. This fact is related to the exploratory behavior of the rats. WKY used as normotensive controls, exhibit standard exploratory behavior and normal levels of physical activity. On the other hand, SHR rats, although hypertensive, may not show significant differences in exploratory behavior and physical activity compared to WKY rats, under normal conditions. These results highlight the importance of investigating the effects of hypertension on memory and provide a basis for future research aimed at better understanding these mechanisms and developing intervention strategies.

This study showed a significantly smaller infarct size in SHR at the 3rd day post-stroke point compared to WKY. However, on the 7th day post-stroke, only WKY rats exhibited a reduction in infarct volume, displaying a smaller infarct size than that in SHR. Previous research has indicated that hypertensive animals have a greater infarct size and more severe motor function sequelae than normotensive animals [43]. A natural reduction in ischemic injury over time, which we observed in WKY rats, involves cells in the penumbra zone where a ‘war zone’ occurs, where healthy cells fight to save those that can still be rescued, reducing the necrotic area [52,53]. Studies emphasize the importance of quickly identifying and treating this region to maximize recovery [54]. However, this reduction was not observed in SHRs. Thus, our results highlight the potential role of infarct size in post-stroke sensorimotor recovery, prompting further investigation into the underlying mechanisms in normotensive and hypertensive rat models.

In this study, we observed that stroke abolished short- and long-term memories in both groups. This may reflect both the extent and the brain region affected by the lesion. As shown in Figure 4, the infarct nucleus reaches the hippocampus, which is the main area related to memory [55]. It is already known that around the infarct nucleus, in the peri-infarct region, the formation of a glial scar can occur, formed by astrocytes and reactive microglia [56,57]. Initially, this scar appears to be beneficial, as it helps contain the heart of the infarction. However, glial scarring may hinder axonal growth in the chronic phase of stroke [56]. From this, we can hypothesize that modulation of neuroinflammation through containment of the infarct core and post-stroke glial scars may allow axonal growth, providing a basis for stroke recovery. This cellular reorganization is one of the most important mechanisms underlying functional recovery and is a potential focus for new therapeutic strategies [58]. Therefore, the peri-infarct region is considered a promising target for new drugs and future therapies associated with hypertension-related acute ischemic stroke.

We encountered some limitations during the execution of this work. One of them was the measurement of the animals’ systemic blood pressure: the equipment, plethysmography, causes a lot of stress to the animals, which can alter systemic blood pressure, generally resulting in higher values than the real ones. The animal needs to remain completely still for the equipment to function properly. However, when placed in the restraint tube, the animal becomes very agitated, compromising the accuracy of the measurement. Additionally, the study was conducted only with males, and we now know that there are differences between genders.

## 5. Conclusions

The results of the present study indicate a negative impact on behavioral outcomes, sensorimotor activity, and long-term memory in animals with SAH. To date, no studies have been found that subjected animals to permanent focal cerebral ischemia using the thermocoagulation model of pial vessels in SHR and WKY rats and evaluated the post-ischemia sensorimotor and memory long-term outcomes. These results reinforce the need to use animal stroke models with comorbidities, such as SAH, to improve translational perspectives for preclinical investigations of the treatment and prevention of stroke in humans.

## Data Availability

All data relevant to this study are available upon request. Please contact the corresponding author to access the data.

## Supplementary Materials

The following supporting information can be downloaded at: https://www.mdpi.com/article/10.3390/brainsci14080838/s1, Table S1: shows the normal distribution of ART results in naïve WKY and WKY ISC animals. Table S2: shows the normal distribution of ART results in naive SHR and SHR ISC animals. Table S3: shows the normal distribution of CT results in naive WKY and naive SHR; WKY ISC and SHR ISC animals. Table S4: shows the normal distribution of OFT results in naive WKY and WKY ISC animals. Table S5: shows the normal distribution of OFT results in naive SHR and SHR ISC animals. The test used was the Shapiro-Wilk test. Figure S1: Symmetry of the front paws of the WKY (a) and SHR (b) groups in the CT. Data expressed individually. Figure S2. Latency of adhesive removal from front paws of WKY and SHR groups. Data expressed individually.

## Abbreviations

ART: Adhesive removal test; CT: Cylinder test; ISC: Ischemic stroke by thermocoagulation; OFT: Open Field Task; SHR: Spontaneously hypertensive rats; SAH: Systemic arterial hypertension; TTC: 2,3,5-triphenyl tetrazolium chloride; WKY: Wistar Kyoto rats.
