# Supplementary material for: Spontaneously Hypertensive Rats Present Exacerbated Focal Stroke Behavioral Outcomes
Source: Brain Sci. 2024 Aug 21;14(8):838. doi: 10.3390/brainsci14080838 (PMC11352869; doi:10.3390/brainsci14080838)
Supplement: Supplementary file 1 [file brainsci-14-00838-s001.zip › brainsci-3123252-supplementary.pdf]

Spontaneously Hypertensive Rats Present Exacerbated Focal Stroke Behavioral Outcomes

João Victor Matos e Moreira[1], Luis Pedro Bernardi[1], Fernanda Cardoso Teixeira[2], Jerônimo Paniago[1], Luciele Varaschini Teixeira[1], Felippo Bifi[1], Diogo Onofre Souza[1], Francieli Rohden[1].

[1]Graduate Program in Biological Sciences: Biochemistry, Universidade Federal do Rio Grande do Sul, Porto Alegre, Rio Grande do Sul, Brazil.

[2] Graduate Program in Biosciences, Federal University of Health Sciences of Porto Alegre - UFCSPA, Porto Alegre, Rio Grande do Sul, Brazil.

Corresponding authors:
1. Diogo Onofre Souza
2. Francieli Rohden

Graduate Program in Biological Sciences: Biochemistry,
Universidade Federal do Rio Grande do Sul,
Ramiro Barcelos Street 2600, annex building
Zip Code: 90035-003
Porto Alegre, Rio Grande do Sul, Brazil.
diogo.bioq@gmail.com or franrohden@gmail.com

Declarations of interest: none

Supplementary Table S1:

**ART - CONTRALATERAL**								
**ART WKY - NAIVE**	**Shapiro-Wilk test**							
N = 5	Day -1	Day 3	Day 7	Day 14	Day 21	Day 28	Day 35	Day 42
P value	0,0541	0,904	0,4537	0,0779	0,1099	0,0809	0,8235	0,8348
Passed normality test (alpha=0.05)?	Yes	Yes	Yes	Yes	Yes	Yes	Yes	Yes
**ART WKY - SHAM**	**Shapiro-Wilk test**							
N = 5	Day -1	*Day 3*	Day 7	Day 14	Day 21	Day 28	Day 35	Day 42
P value	0,0629	0,9489	0,4899	0,1342	0,5225	0,9676	0,7466	0,5663
Passed normality test (alpha=0.05)?	Yes	Yes	Yes	Yes	Yes	Yes	Yes	Yes
**ART WKY - ISC**	**Shapiro-Wilk test**							
N = 10	Day -1	*Day 3*	Day 7	Day 14	Day 21	Day 28	Day 35	Day 42
P value	0,0367	0,8263	0,0048	0,7846	0,0188	0,2701	0,1093	0,6236
Passed normality test (alpha=0.05)?	No	Yes	No	Yes	No	Yes	Yes	Yes

**ART - IPSILATERAL**								
**ART WKY - NAIVE**	**Shapiro-Wilk test**							
N = 5	Day -1	Day 3	Day 7	Day 14	Day 21	Day 28	Day 35	Day 42
P value	0,6985	0,8083	0,1783	0,2234	0,0903	0,4537	0,217	0,3522
Passed normality test (alpha=0.05)?	Yes	Yes	Yes	Yes	Yes	Yes	Yes	Yes
**ART WKY - SHAM**	**Shapiro-Wilk test**							
N = 5	Day -1	*Day 3*	Day 7	Day 14	Day 21	Day 28	Day 35	Day 42
P value	0,5001	0,4931	0,7577	0,5297	0,8961	0,3562	0,4951	0,4399
Passed normality test (alpha=0.05)?	Yes	Yes	Yes	Yes	Yes	Yes	Yes	Yes
**ART WKY - ISC**	**Shapiro-Wilk test**							
N = 10	Day -1	*Day 3*	Day 7	Day 14	Day 21	Day 28	Day 35	Day 42
P value	0,7612	0,2808	0,2851	0,9403	0,1702	0,7638	0,0601	0,3318
Passed normality test (alpha=0.05)?	Yes	Yes	Yes	Yes	Yes	Yes	Yes	Yes

Supplementary Table S1: This table shows the normal distribution of ART results in naïve WKY and WKY ISC animals. The test used was the Shapiro-Wilk test.

Supplementary Table S2:

ART - CONTRALATERAL								
**ART SHR - NAIVE**	**Shapiro-Wilk test**							
N = 5	Day -1	Day 3	Day 7	Day 14	Day 21	Day 28	Day 35	Day 42
P value	0,4703	0,1336	0,4105	0,3316	0,124	0,5298	0,7417	0,858
Passed normality test (alpha=0.05)?	Yes	Yes	Yes	Yes	Yes	Yes	Yes	Yes
**ART SHR - SHAM**	**Shapiro-Wilk test**							
N = 5	Day -1	*Day 3*	Day 7	Day 14	Day 21	Day 28	Day 35	Day 42
P value	0,1422	0,9775	0,3334	0,5273	0,4352	0,1034	0,4087	0,5609
Passed normality test (alpha=0.05)?	Yes	Yes	Yes	Yes	Yes	Yes	Yes	Yes
**ART SHR - ISC**	**Shapiro-Wilk test**							
N = 10	Day -1	*Day 3*	Day 7	Day 14	Day 21	Day 28	Day 35	Day 42
P value	0,7733	0,0624	0,0549	0,1029	0,0613	0,4931	0,8255	0,5708
Passed normality test (alpha=0.05)?	Yes	Yes	Yes	Yes	Yes	Yes	Yes	Yes

ART - IPSILATERAL								
**ART SHR - NAIVE**	**Shapiro-Wilk test**							
N = 5	Day -1	Day 3	Day 7	Day 14	Day 21	Day 28	Day 35	Day 42
P value	0,5288	0,9985	0,4442	0,2693	0,2001	0,2647	0,1618	0,6275
Passed normality test (alpha=0.05)?	Yes	Yes	Yes	Yes	Yes	Yes	Yes	Yes
**ART SHR - SHAM**	**Shapiro-Wilk test**							
N = 5	Day -1	*Day 3*	Day 7	Day 14	Day 21	Day 28	Day 35	Day 42
P value	0,05	0,9269	0,1795	0,9719	0,776	0,3507	0,1995	0,4211
Passed normality test (alpha=0.05)?	Yes	Yes	Yes	Yes	Yes	Yes	Yes	Yes
**ART SHR - ISC**	**Shapiro-Wilk test**							
N = 10	Day -1	*Day 3*	Day 7	Day 14	Day 21	Day 28	Day 35	Day 42
P value	0,2059	0,7195	0,4113	0,1878	0,0299	0,3001	0,4185	0,4748
Passed normality test (alpha=0.05)?	Yes	Yes	Yes	Yes	No	Yes	Yes	Yes

Supplementary Table S2: This table shows the normal distribution of ART results in naive SHR and SHR ISC animals. The test used was the Shapiro-Wilk test.

Supplementary Table S3:

**CT WKY - ISC**	**Shapiro-Wilk test**							
N = 10	Day -1	Day 3	Day 7	Day 14	Day 21	Day 28	Day 35	Day 42
P value	0,5952	0,5466	0,0956	0,5489	0,4199	0,6247	0,4103	0,69
Passed normality test (alpha=0.05)?	Yes	Yes	Yes	Yes	Yes	Yes	Yes	Yes

**CT SHR - ISC**	**Shapiro-Wilk test**							
N = 12	Day -1	*Day 3*	Day 7	Day 14	Day 21	Day 28	Day 35	Day 42
P value	0,9004	*0,0523*	0,1093	0,3003	0,062	0,9987	0,7721	0,0876
Passed normality test (alpha=0.05)?	Yes	Yes	Yes	Yes	Yes	Yes	Yes	Yes

Supplementary Table S3: This table shows the normal distribution of CT results in naive WKY and naive SHR; WKY ISC and SHR ISC animals. The test used was the Shapiro-Wilk test.

Supplementary Table S4

**OFT - 1ª exp**										
**WKY - NAIVE**	**Shapiro-Wilk test**									
N = 12	1'	2'	3'	4'	5'	6'	7'	8'	9'	10'
P value	0,459	0,660	0,689	0,222	0,155	0,535	0,672	0,848	0,318	0,544
Passed normality test (alpha=0.05)?	Yes	Yes	Yes	Yes	Yes	Yes	Yes	Yes	Yes	Yes

**OFT - 2ª exp**										
**WKY - NAIVE**	**Shapiro-Wilk test**									
N = 12	1'	2'	3'	4'	5'	6'	7'	8'	9'	10'
P value	0,293	0,610	0,325	0,342	0,724	0,588	0,504	0,917	0,211	0,248
Passed normality test (alpha=0.05)?	Yes	Yes	Yes	Yes	Yes	Yes	Yes	Yes	Yes	Yes

**OFT - 1ª exp**										
**WKY - ISC**	**Shapiro-Wilk test**									
N = 12	1'	2'	3'	4'	5'	6'	7'	8'	9'	10'
P value	0,702	0,703	0,636	0,731	0,153	0,708	0,625	0,245	0,529	0,492
Passed normality test (alpha=0.05)?	Yes	Yes	Yes	Yes	Yes	Yes	Yes	Yes	Yes	Yes

**OFT - 2ª exp**										
**WKY - ISC**	**Shapiro-Wilk test**									
N = 12	1'	2'	3'	4'	5'	6'	7'	8'	9'	10'
P value	0,623	0,475	0,079	0,279	0,768	0,170	0,437	0,416	0,052	0,608
Passed normality test (alpha=0.05)?	Yes	Yes	Yes	Yes	Yes	Yes	Yes	Yes	Yes	Yes

Supplementary Table S4: This table shows the normal distribution of OFT results in naive WKY and WKY ISC animals. The test used was the Shapiro-Wilk test.

Supplementary Table S5:

OFT - 1ª exp										
SHR - NAIVE	Shapiro-Wilk test									
N = 12	1'	2'	3'	4'	5'	6'	7'	8'	9'	10'
P value	0,937	0,702	0,771	0,972	0,008	0,179	0,653	0,490	0,722	0,933
Passed normality test (alpha=0.05)?	Yes	Yes	Yes	Yes	Yes	Yes	Yes	Yes	Yes	Yes

OFT - 2ª exp										
SHR - NAIVE	Shapiro-Wilk test									
N = 12	1'	2'	3'	4'	5'	6'	7'	8'	9'	10'
P value	0,310	0,406	0,410	0,656	0,176	0,253	0,295	0,907	0,819	0,893
Passed normality test (alpha=0.05)?	Yes	Yes	Yes	Yes	Yes	Yes	Yes	Yes	Yes	Yes

OFT - 1ª exp										
SHR - ISC	Shapiro-Wilk test									
N = 12	1'	2'	3'	4'	5'	6'	7'	8'	9'	10'
P value	0,951	0,945	0,980	0,934	0,939	0,931	0,931	0,893	0,946	0,938
Passed normality test (alpha=0.05)?	Yes	Yes	Yes	Yes	Yes	Yes	Yes	Yes	Yes	Yes

OFT - 2ª exp										
SHR - ISC	Shapiro-Wilk test									
N = 12	1'	2'	3'	4'	5'	6'	7'	8'	9'	10'
P value	0,976	0,283	0,344	0,262	0,889	0,153	0,350	0,411	0,090	0,225
Passed normality test (alpha=0.05)?	Yes	Yes	Yes	Yes	Yes	Yes	Yes	Yes	Yes	Yes

Supplementary Table S5: This table shows the normal distribution of OFT results in naive SHR and SHR ISC animals. The test used was the Shapiro-Wilk test.

Supplementary Figure S1:

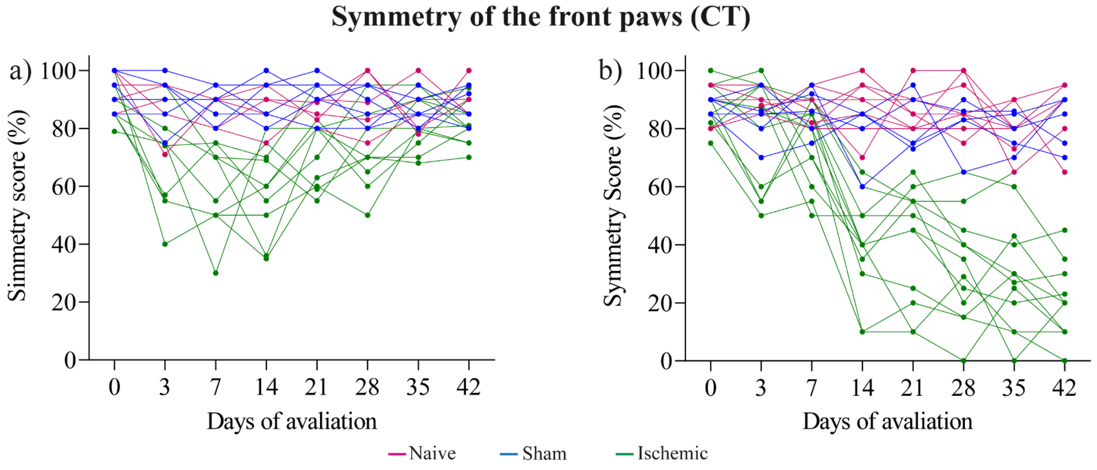

Supplementary Figure S1: Symmetry of the front paws of the WKY (a) and SHR (b) groups in the CT. Data expressed individually.

Supplementary Figure S2:

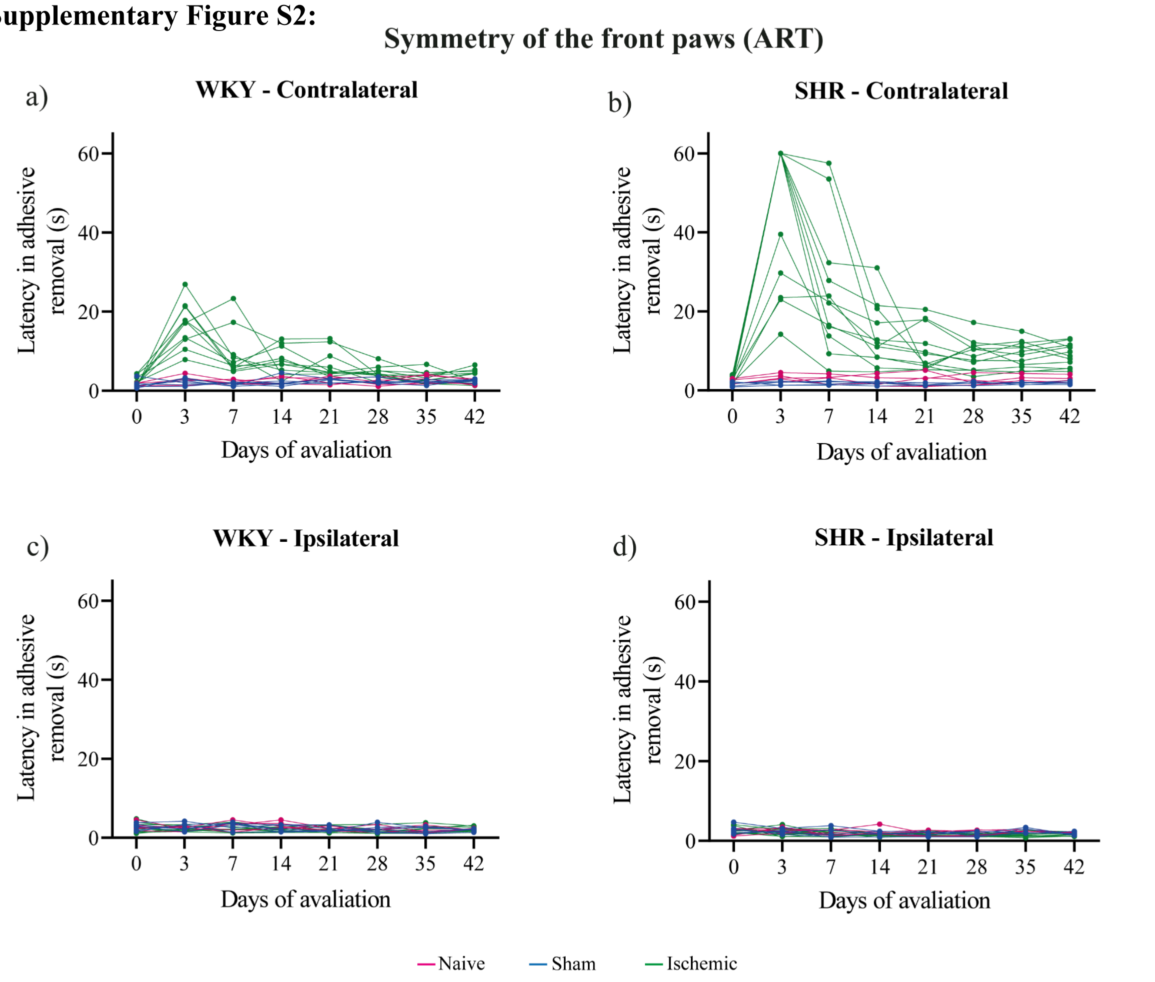

Supplementary Figure S2. Latency of adhesive removal from front paws of WKY (**Figures a and c**) and SHR (**Figures b and d**) groups. Data expressed individually.
